# Severe hyperandrogenism and mild autonomous cortisol secretion from a functional lipid-poor adrenal cortical adenoma

**DOI:** 10.1210/jcemcr/luag059

**Published:** 2026-04-08

**Authors:** Elio Monsour, Prerna Dogra, Stephanie Giparas, William H Westra, Rachel Voss, Colleen Veloski

**Affiliations:** Department of Head and Neck-Endocrine Oncology, Moffitt Cancer Center, Tampa, FL 33612, USA; Department of Head and Neck-Endocrine Oncology, Moffitt Cancer Center, Tampa, FL 33612, USA; Department of Head and Neck-Endocrine Oncology, Moffitt Cancer Center, Tampa, FL 33612, USA; Department of Pathology, Moffitt Cancer Center, Tampa, FL 33612, USA; Department of Sarcoma Oncology, Moffitt Cancer Center, Tampa, FL 33612, USA; Department of Head and Neck-Endocrine Oncology, Moffitt Cancer Center, Tampa, FL 33612, USA

**Keywords:** adrenal mass, hyperandrogenism, cortisol secretion, lipid-poor adrenal adenoma, adrenalectomy

## Abstract

A 44-year-old woman presented with progressive hirsutism, deepening of voice, irregular menses, and left flank discomfort. Laboratory evaluation revealed markedly elevated serum testosterone and androstenedione levels, along with unsuppressed cortisol following an overnight 1 mg dexamethasone test. Notably, dehydroepiandrosterone sulfate (DHEA-S) levels remained within normal limits. Imaging identified a 4.2-cm heterogeneously enhancing left adrenal mass with a precontrast attenuation of 15 Hounsfield units, consistent with a lipid-poor lesion. 18F-fluorodeoxyglucose positron emission tomography-computed tomography demonstrated intensely avid uptake. Open adrenalectomy was performed, and final pathology confirmed a benign adrenocortical adenoma. Postoperatively, both testosterone and androstenedione levels normalized. The patient received temporary hydrocortisone replacement per our institution's protocol for treatment of presumed postoperative adrenal insufficiency, which was discontinued after biochemical recovery of endogenous cortisol production. This case highlights that not all androgen and cortisol co-secreting adrenal masses are malignant, and that preoperative normal DHEA-S levels do not exclude an underlying adrenal source of hyperandrogenism.

## Introduction

Adrenal tumors co-secreting androgens and cortisol are uncommon but clinically significant, with the majority representing adrenocortical carcinoma (ACC) [1]. Early recognition is critical for timely and appropriate management. Historically, these tumors have been considered rare, but with increasing comprehensive endocrine evaluation in patients with adrenal nodules, the prevalence is likely higher than previously recognized. While cortisol and aldosterone-producing adenomas are relatively well characterized, tumors with mixed steroidogenic profiles are rare and not as well understood. In a nationwide survey of adrenal incidentalomas in Japan, the authors found that among over 3000 cases, only 0.2% involved androgen-producing tumors, and a subset of these tumors also exhibited cortisol co-secretion, highlighting the rarity of this presentation and the need for careful hormonal evaluation in patients with virilization [2]. Herein, we describe the case of a middle-aged woman who presented with marked hyperandrogenism and was ultimately found to have a lipid-poor adrenal cortical adenoma secreting both androgens and cortisol.

## Case presentation

A 44-year-old woman presented for evaluation of a left adrenal mass identified during evaluation of progressive hirsutism, deepening of voice, and irregular menses over the preceding 6 months. She reported shaving facial and chest hair every 3 to 4 days. She noted a reduction in breast tissue and associated weight loss attributed to increased physical activity. She denied exogenous androgen exposure. She had no history of hypertension, electrolyte disturbances, diabetes mellitus, fragility fractures, venous thromboembolism, or glucocorticoid use aside from intermittent nasal fluticasone. She had no family history of adrenal or endocrine tumors. On exam, she was normotensive and well-appearing, without cushingoid features such as facial rounding, striae, or proximal muscle weakness. She had moderate-to-severe hirsutism, with a Ferriman-Gallwey score of 18, including coarse hair on the chin, upper lip, chest, and lower abdomen. There was no acne, temporal balding, or evidence of muscle hypertrophy. Breast tissue was reduced but symmetric.

## Diagnostic assessment

**Figure 1:**
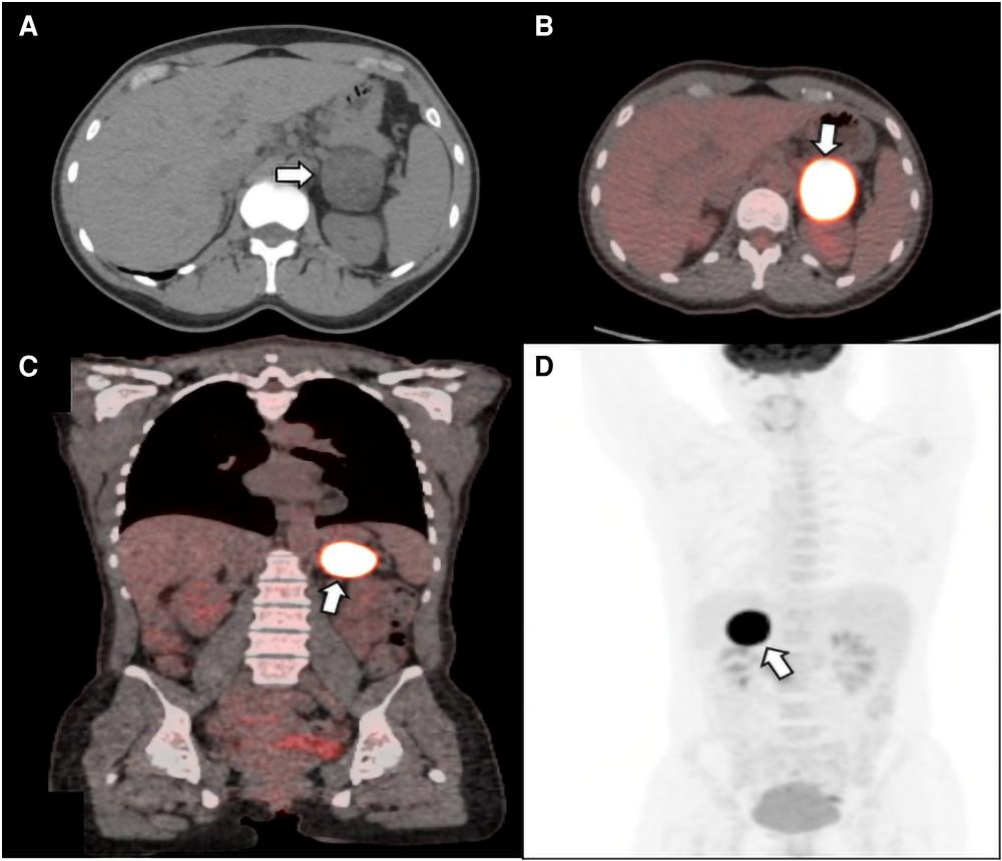
Axial computed tomography of the abdomen (A) of the same patient before intravenous contrast administration shows a 4.3 cm heterogeneous left adrenal mass (white arrow) with an unenhanced attenuation value of around 15 HU. On ^18^FDG-PET images (B-D), the lesion demonstrates avid ^18^FDG uptake with a with SUV maximum 38.7. Abbreviations: HU, Hounsfield units; ^18^FDG-PET, 18F-fluorodeoxyglucose positron emission tomography; SUV, standardized uptake value.

Laboratory testing revealed elevated total and free testosterone, elevated androstenedione, and normal dehydroepiandrosterone sulfate (DHEA-S) and prolactin levels (Table 1). The baseline morning adrenocorticotropic hormone (ACTH) level was low-normal, and the overnight 1-mg dexamethasone suppression test showed an unsuppressed cortisol level of 6.9 μg/dL (SI: 190.44 nmol/L) (reference range, ≤1.8 μg/dL [SI: ≤49.68 nmol/L]), with an optimal serum dexamethasone level indicating autonomous cortisol production. Transvaginal ultrasound was unrevealing. Computed tomography (CT) abdominal imaging demonstrated a 4.2-cm left adrenal mass with heterogeneous enhancement and a precontrast attenuation of 15 Hounsfield units (HU), consistent with a lipid-poor lesion (Fig. 1A). 18F-fluorodeoxyglucose positron emission tomography-computed tomography (18F-FDG PET-CT) revealed intensely avid uptake in the left adrenal mass. (Fig. 1B-1D).

**Table 1:** Key endocrine laboratory findings pre- and postadrenalectomy

Laboratory test	At presentation	At 4-week follow-up	Reference range
Total testosterone	365 ng/dL (12.67 nmol/L)	14.17 ng/dL (0.51 nmol/L)	2-45 ng/dL (0.07-1.56 nmol/L)
Free testosterone	43.4 pg/mL (150.60 pmol/L)	1.1 pg/mL (3.82 pmol/L)	1.1-5.8 pg/mL (3.82-20.13 pmol/L)
A4	2793 ng/dL (97.48 nmol/L)	63 ng/dL (2.20 nmol/L)	30-200 ng/dL (1.05-6.98 nmol/L)
DHEA-S	171 µg/dL (4.63 mmol/L)	—	35-430 µg/dL (0.95-11.65 mmol/L)
17-Hydroxyprogesterone	813 ng/dL (24.63 nmol/L)	81.3 ng/dL (2.46 nmol/L)	≤206 ng/dL (≤6.24 nmol/L)
ACTH	15 pg/mL (3.3 pmol/L)	45.1 pg/mL (9.92 pmol/L)	7.2-63.3 pg/mL (1.58-13.93 pmol/L)
11-Deoxycortisol	12 ng/dL (0.35 nmol/L)	—	3-40 ng/dL (0.087-1.16 nmol/L)
Cortisol after 1-mg DST	6.9 µg/dL (190.44 nmol/L)	—	≤1.8 µg/dL (≤49.68 nmol/L)
Dexamethasone level	456 ng/dL (11 618.88 pmol/L)	Not applicable	180-550 ng/dL (4586.4-14,014 pmol/L)
Baseline cortisol by LC/MS	10.5 µg/dL (289.8 nmol/L)	11.7 µg/dL (322.92 nmol/L)	4.6-20.6 µg/dL (126.96-568.56 nmol/L)
E2	116 pg/mL (425.72 pmol/L)	—	Follicular phase: 19-144 pg/mL (69.73-528.48 pmol/L)
PRL	21.3 ng/mL (0.93 nmol/L)	—	Non-pregnant: 3-30 ng/mL (0.13-1.30 nmol/L)
HgbA1C	5.2% (33 mmol/mol)	—	<5.7% (<39 mmol/mol)
Plasma Metanephrines	144 pg/mL (757.44 pmol/L)	—	≤205 pg/mL (≤1078.3 pmol/L)

Abbreviations: A4, androstenedione; ACTH, adrenocorticotropic hormone; DHEA-S, dehydroepiandrosterone sulfate; DST, dexamethasone suppression test; E2, estradiol; LC/MS, liquid chromatography-mass spectrometry; PRL, prolactin.

## Treatment

**Figure 2:**
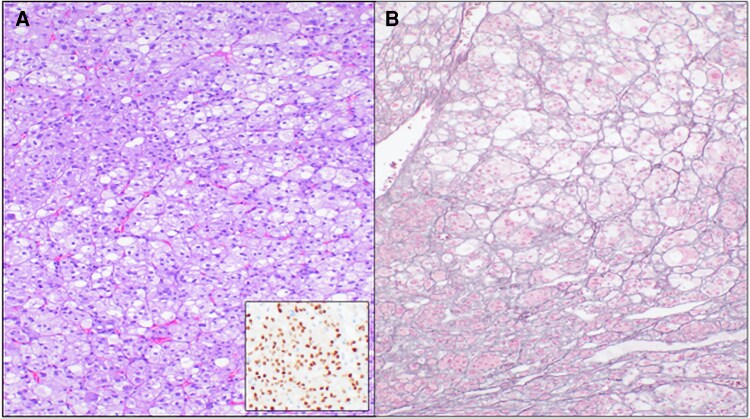
Adrenal cortical adenoma characterized by cells with abundant eosinophilic (lipid-poor) to vacuolated (lipid-rich) cytoplasm growing in a nested pattern (A, hematoxylin eosin stain, 10 × magnification). The benign nature of the tumor is supported by a preserved reticulin framework (B, reticulin stain, 10 × magnification) and absence of necrosis and prominent mitotic activity. The adrenal cortical nature of the tumor is confirmed by nuclear expression of steroidogenic Factor 1 (A inset, SF1 immunohistochemistry, 20 × magnification).

Given concern for possible ACC, the patient underwent open left adrenalectomy with periadrenal retroperitoneal lymphadenectomy. Gross pathology showed a well-circumscribed, noninvasive tumor. Histopathology confirmed an adrenal cortical adenoma positive for steroidogenic factor 1 with a Ki-67 proliferation rate of 5%. There was no necrosis, capsular, or vascular invasion identified. There were no malignant criteria present using the Weiss classification scheme (Weiss score: 0) [3, 4]. (Fig. 2)

Although traditionally, androgen and cortisol adrenal co-secreting adrenal tumors are associated with ACC, emerging reports of benign adrenal adenomas with mixed steroidogenic profile necessitate the need for individualized management regarding the optimal and most appropriate surgical approach.

## Outcome and follow-up

Postoperatively, the patient was placed on hydrocortisone for presumed postoperative adrenal insufficiency based on our institution's protocol. She did not exhibit clinical signs or symptoms of adrenal crisis. Repeat laboratory testing at 1-month follow-up showed normalization of total testosterone, free testosterone, 17-hydroxyprogesterone, and androstenedione levels (Table 1). Additionally, morning serum cortisol was normal off hydrocortisone and glucocorticoid replacement was discontinued.

## Discussion

Adrenal cortical tumors exclusively secreting androgens are an uncommon cause of hyperandrogenism [2]. Functional adrenal neoplasms that co-secrete both androgens and cortisol add to the complexity of clinical evaluation and management. In this case, the patient exhibited severe hyperandrogenism with progressive hirsutism, deepening of the voice, and irregular menses, all suggestive of a tumor source given the rapid onset and degree of virilization.

Although DHEA-S is frequently used as a biomarker of adrenal androgen production, normal DHEA-S levels do not exclude an adrenal etiology, particularly in tumors predominantly secreting testosterone or androstenedione. In a cohort study by Arlt et al, normal DHEA-S concentrations were noted in approximately 20-30% of patients with adrenal androgen-producing tumors [5]. One potential explanation for the discordance between elevated downstream androgens and normal DHEA-S levels is that certain adrenal tumors may bypass early ACTH-dependent steps in steroidogenesis, preferentially synthesizing androstenedione and testosterone through upregulation of enzymes such as 3β-hydroxysteroid dehydrogenase (3β-HSD) and 17β-hydroxysteroid dehydrogenase (17β-HSD), while producing minimal DHEA or DHEA-S [6]. In tumors that co-secrete cortisol, even mild autonomous cortisol production, as evidenced in this case by an abnormal 1-mg dexamethasone suppression test, may lead to partial suppression of ACTH, further attenuating DHEA-S synthesis [7]. This highlights the importance of evaluating the full androgen panel, rather than relying solely on DHEA-S, when adrenal hyperandrogenism is suspected.

Imaging characteristics also play a pivotal role in assessment. Lipid-poor adrenal masses (defined as precontrast HU >10) are more likely to be malignant [8]. In this case, the lesion's lipid-poor nature combined with PET avidity (SUV max 38) raised significant concern for adrenal cortical carcinoma [9]. However, definitive histopathologic evaluation revealed a benign adrenal cortical adenoma, characterized by circumscribed growth, low mitotic activity, preserved reticulin framework, and absence of necrosis or vascular invasion [10].

Adrenal cortical carcinoma must always be considered in the differential diagnosis of hyperandrogenic adrenal tumors, particularly when radiologic features are suspicious. However, benign cortisol and androgen cosecreting adenomas have been described, with at least 10 cases reported in the literature [11]. These tumors typically exhibit milder autonomous cortisol secretion compared to overt Cushing syndrome, yet postoperative adrenal insufficiency is common due to suppression of the contralateral adrenal axis.

This case underscores the critical importance of a multidisciplinary approach, incorporating biochemical, radiologic, and histopathologic data, to differentiate between benign and malignant adrenal pathology. Furthermore, it reinforces the need for postoperative endocrine follow-up, as even subclinical cortisol secretion preoperatively may predispose patients to clinically significant adrenal insufficiency postadrenalectomy. In summary, this case adds to the limited literature on dual-secreting adrenal cortical adenomas, highlights the diagnostic pitfalls associated with normal DHEA-S levels, and emphasizes the necessity of comprehensive evaluation in women with rapidly progressive hyperandrogenism.

## Learning points

- Cortisol and androgen cosecreting adrenal cortical adenomas are exceptionally rare.
- Normal DHEA-S does not exclude adrenal hyperandrogenism as some adrenal tumors preferentially produce androstenedione and testosterone via alternative steroidogenic pathways.
- Identifying subclinical cortisol excess preoperatively allows for anticipatory planning of postoperative glucocorticoid replacement.
- Radiologic suspicion does not always indicate malignancy but lipid poor, FDG avid adrenal masses can mimic malignancy. Definitive diagnosis requires histopathology.

## Contributors

All authors made individual contributions to authorship. E.M. and S.G. were involved in the clinical diagnosis and longitudinal management of the patient. P.D. and C.V. contributed to data acquisition, literature review, and drafting of the manuscript. W.H.W. was involved in the histopathology section and the preparation of histology images. R.V. performed the surgical procedure and contributed to perioperative management and manuscript revision. All authors approved the final manuscript.

## Data Availability

Data sharing does not apply to this article as no datasets were generated or analyzed during the current study.
